# Food for Teething Children

**Published:** 1875-09

**Authors:** 


### FOOD FOR TEETHING CHILDREN.

Teething children who are beginning to eat solid food, can be supplied with nothing better than the Granulated Wheat Biscuit. The child will not attempt to swallow this food until it is softened by mastication, and the mechanical action of the biscuit upon the gums will greatly assist the teeth in their eruption. The act of eating this food will necessarily occupy much time, and this will give the teeth and jaws considerable valuable exercise. The food thus swallowed proves very nutritious, and rapidly builds up small boys and girls, as well as larger ones. In all stomach troubles and bowel complaints, they seem to have wonderful power to regulate and restore.

---

ANCIENT AND MODERN.—With the use of modern machinery, a single miller is now able to produce flour enough for the daily supply of 3,600 men. With the hand-mill, which was the machine for grinding grain among the Greeks, a man could produce only flour enough to supply 25 men. Thus the machinery of the present mill represents the labor of 144 men. A lively knitter can make 30 stitches a minute with her kneedles. The knitting-machine will make 4,800 stitches in the same time; or, in other words, works with an efficiency equivalent to 160 laborers. In the light of these illustrations, if we compare the slow process of hand labor which prevailed among the ancients, 1,000 years after Homer's time, with the abundant and complex luxuries enjoyed by the aristocratic and idle classes of that period, we shall give some conception of the drudgery performed by the laboring people, and of their miserable reward. The splendor of antiquity grows dull and unenviable when it is considered at what a vast expense of human poverty and suffering it was procured.

